# Supplementary material for: Forest fires and climate-induced tree range shifts in the western US
Source: Nat Commun. 2021 Nov 15;12:6583. doi: 10.1038/s41467-021-26838-z (PMC8594433; doi:10.1038/s41467-021-26838-z)
Supplement: Supplementary file 1 — Supplementary Information [file 41467_2021_26838_MOESM1_ESM.pdf]

Supplementary Tables

Results with and without Niche Difference Direction Filtering

	Schoener's D		Centroid Distance		Difference in Centroid Distance (CD^B - CD^U)	
	Burned	Unburned	Burned	Unburned	Mean	95% CI
**Filtered By:<br>• Life-Stage<br>• Fire**						
Chrysolepis chrysophylla	0.518*	0.641*	0.529	0.677*	-0.148	
Picea engelmannii	0.369*	0.751*	0.508†	0.375*	0.132	
Pinus albicaulis	0.32	0.768*	0.294	0.234*	0.06	
Pinus contorta	0.534	0.692*	0.308	0.314*	-0.006	
Pinus ponderosa	0.498	0.68*	0.616*	0.198*	0.418	
Pseudotsuga menziesii	0.421*	0.704*	1.095*	0.592*	0.504*	
Quercus chrysolepis	0.688*	0.852*	0.714*	0.282*	0.432*	
Quercus kelloggii	0.574	0.825*	0.641†	0.194*	0.447	
**Filtered By:<br>• Life-Stage**						
Abies grandis	0.443	0.683*	0.206	0.195*	0.011	
Arbutus menziesii	0.512*	0.752*	0.126	0.462*	-0.336	
Calocedrus decurrens	0.125†	0.778*	0.48	0.343*	0.137	
Chrysolepis chrysophylla	0.518*	0.641*	0.529	0.677*	-0.148	
Lithocarpus densiflorus	0.688*	0.734*	0.683*	0.383*	0.3†	
Picea engelmannii	0.369*	0.751*	0.508†	0.375*	0.132	
Pinus albicaulis	0.32	0.768*	0.294	0.234*	0.06	
Pinus contorta	0.534	0.692*	0.308	0.314*	-0.006	
Pinus ponderosa	0.498	0.68*	0.616*	0.198*	0.418	
Pseudotsuga menziesii	0.421*	0.704*	1.095*	0.592*	0.504*	
Quercus chrysolepis	0.688*	0.852*	0.714*	0.282*	0.432*	
Quercus kelloggii	0.574	0.825*	0.641†	0.194*	0.447	
**Filtered By:<br>• Fire**						
Abies lasiocarpa	0.55	0.814*	0.314	0.151*	0.163	
Chrysolepis chrysophylla	0.518*	0.641*	0.529	0.677*	-0.148	
Larix occidentalis	0.546	0.742†	0.254	0.091†	0.164	
Picea engelmannii	0.369*	0.751*	0.508†	0.375*	0.132	
Pinus albicaulis	0.32	0.768*	0.294	0.234*	0.06	
Pinus contorta	0.534	0.692*	0.308	0.314*	-0.006	
Pinus monticola	0.132*	0.845*	2.562*	0.326*	2.236*	
Pinus ponderosa	0.498	0.68*	0.616*	0.198*	0.418	
Pseudotsuga menziesii	0.421*	0.704*	1.095*	0.592*	0.504*	
Quercus chrysolepis	0.688*	0.852*	0.714*	0.282*	0.432*	
Quercus gambelii	0.638	0.69*	0.425	0.406*	0.019	
Quercus kelloggii	0.574	0.825*	0.641†	0.194*	0.447	
Umbellularia californica	0.484*	0.741*	1.755*	0.38*	1.375*	
**Not Filtered**						
Abies concolor	0.496	0.755*	0.616	0.173*	0.443	
Abies grandis	0.443	0.683*	0.206	0.195*	0.011	
Abies lasiocarpa	0.55	0.814*	0.314	0.151*	0.163	
Acer macrophyllum	0.332†	0.85	0.7	0.252*	0.448	
Arbutus menziesii	0.512*	0.752*	0.126	0.462*	-0.336	
Calocedrus decurrens	0.125†	0.778*	0.48	0.343*	0.137	
Chrysolepis chrysophylla	0.518*	0.641*	0.529	0.677*	-0.148	
Cornus nuttallii	0.354*	0.774*	0.84	0.297*	0.543	
Juniperus occidentalis	0.494	0.652*	0.271	0.142*	0.129	
Larix occidentalis	0.546	0.742†	0.254	0.091†	0.164	
Lithocarpus densiflorus	0.688*	0.734*	0.683*	0.383*	0.3†	
Picea engelmannii	0.369*	0.751*	0.508†	0.375*	0.132	
Pinus albicaulis	0.32	0.768*	0.294	0.234*	0.06	
Pinus contorta	0.534	0.692*	0.308	0.314*	-0.006	
Pinus lambertiana	0.436	0.809*	0.443	0.278*	0.165	
Pinus monticola	0.132*	0.845*	2.562*	0.326*	2.236*	
Pinus ponderosa	0.498	0.68*	0.616*	0.198*	0.418	
Populus tremuloides	0.606*	0.807*	0.398*	0.166*	0.232*	
Prunus emarginata	0.066*	0.718*	0.812	0.693*	0.119	
Pseudotsuga menziesii	0.421*	0.704*	1.095*	0.592*	0.504*	
Quercus chrysolepis	0.688*	0.852*	0.714*	0.282*	0.432*	
Quercus gambelii	0.638	0.69*	0.425	0.406*	0.019	
Quercus garryana	0.221	0.714*	0.622*	0.446*	0.176	
Quercus kelloggii	0.574	0.825*	0.641†	0.194*	0.447	
Quercus wislizeni	0.145*	0.744	0.926*	0.13	0.795*	
Umbellularia californica	0.484*	0.741*	1.755*	0.38*	1.375*	

Supplementary Table 1 Results table showing the primary climatic niche distance analysis under different species vetting scenarios. Values appended with * or † indicate statistically significant evidence of SORD ($p < .05$ and $p < .1$, respectively), where the null hypothesis of no SORD corresponds to Schoener's D = 1 and Centroid Distance = 0. The results from species where the direction of SORDs are vetted between both life-stage and burned-unburned groups are presented in the manuscript. Results from the other groups (life-stage-only filtering, fire-only filtering, and no filtering) confirm that the observation that fire increased SORD is consistent across analyses with variable degrees of species vetting.

Collinearity Analysis that Determined Climate Variable Selection

Climate Variable	Mean VIF
Mean Summer Precipitation	1.6
Mean Winter Precipitation	2.42
Mean Temperature of Coldest Month	4.51
Mean Temperature of Warmest Month	4.67
Potential Evapotranspiration	5.83
Growing Degree Days	10 <
Mean Annual Precipitation	10 <
Mean Annual Temperature	10 <
Actual / Potential Evapotranspiration	10 <

Supplementary Table 2 Results of collinearity analysis using vifstep() function from *usdm* package. The 8 initial climatic variables recommended for this type of analysis were Mean Temperature of the Coldest Month, Mean Temperature of the Warmest Month, Mean Annual Precipitation, Ratio of Actual to Potential Evapotranspiration, Potential Evapotranspiration, Precipitation Seasonality, Mean Annual Temperature, and Growing Degree Days[1,2]. We supplanted Precipitation Seasonality with Mean Summer Precipitation and Mean Winter Precipitation. We determined collinearity of the 9 variables by running the vifstep() function (which incrementally excluded variables with a VIF greater than 10) of the *usdm* R package[3], for 100 iterations. We then averaged the resulting VIFs of each iteration to produce Table A3. Of the 5 climatic variables with VIF < 10, we chose the 4 with the least collinearity for further analysis: Mean Summer Precipitation, Mean Winter Precipitation, Mean Temperature of Coldest Month, and Mean Temperature of Warmest Month.

	Schoener's D		Centroid Distance		Difference in Centroid Distance (CD^B - CD^U)	
	Burned	Unburned	Burned	Unburned	Mean	95% CI
Chrysolepis chrysophylla	0.528*	0.66*	0.401	0.558*	-0.158	
Pinus ponderosa	0.493*	0.707*	0.581*	0.175*	0.406†	
Pseudotsuga menziesii	0.437*	0.693*	0.866*	0.5*	0.365	
Quercus chrysolepis	0.724*	0.848*	0.55*	0.189*	0.361*	
Quercus kelloggii	0.617	0.784*	0.324	0.1*	0.224	

Supplementary Table 3.1 Results table for reanalysis with Mean Temperature of the Coldest Month, Mean Summer Precipitation, Mean Winter Precipitation. Values appended with * indicate statistical significance ($p < .05$), and † denotes $p < .1$. Schoener's D and Centroid Distance were tested for statistical significance using the one-sided niche equivalency test and two-sided Hotelling's T2 Test, respectively (*Chrysolepis chrysophylla*: $p_{Schoener's\ D,\ Burned} = .0279$, $p_{Schoener's\ D,\ Unburned} = .00398$, $p_{Centroid\ Distance,\ Burned} = .358$, $p_{Centroid\ Distance,\ Unburned} < 2.20e^{-16}$; *Pinus ponderosa*: $p_{Schoener's\ D,\ Burned} = .0359$, $p_{Schoener's\ D,\ Unburned} = .00398$, $p_{Centroid\ Distance,\ Burned} = 5.75e^{-5}$, $p_{Centroid\ Distance,\ Unburned} = 9.19e^{-10}$; *Pseudotsuga menziesii*: $p_{Schoener's\ D,\ Burned} = .00797$, $p_{Schoener's\ D,\ Unburned} = .00398$, $p_{Centroid\ Distance,\ Burned} = .00255$, $p_{Centroid\ Distance,\ Unburned} < 2.20e^{-16}$; *Quercus chrysolepis*: $p_{Schoener's\ D,\ Burned} = .0159$, $p_{Schoener's\ D,\ Unburned} = .00398$, $p_{Centroid\ Distance,\ Burned} = 4.97e^{-5}$, $p_{Centroid\ Distance,\ Unburned} = 4.84e^{-7}$; *Quercus kelloggii*: $p_{Schoener's\ D,\ Burned} = .773$, $p_{Schoener's\ D,\ Unburned} = .00797$, $p_{Centroid\ Distance,\ Burned} = .262$, $p_{Centroid\ Distance,\ Unburned} = .0310$). $CD_B - CD_U$ is the result of subtracting the Centroid Distance in unburned samples by the Centroid Distance in burned samples, where the null hypothesis is that $CD_B - CD_U = 0$ and wildfire occurrence does not impact SORD. Statistical significance for this metric was calculated using a two-sided bootstrap test. The sample sizes used to produce these metrics are found in Table 1.

	Schoener's D		Centroid Distance		Difference in Centroid Distance (CD^B - CD^U)	
	Burned	Unburned	Burned	Unburned	Mean	95% CI
Chrysolepis chrysophylla	0.684	0.645*	0.478	0.54*	-0.062	
Picea engelmannii	0.402*	0.749*	0.49†	0.355*	0.135	
Pinus contorta	0.55	0.708*	0.268	0.27*	-0.002	
Pinus ponderosa	0.487	0.653*	0.533*	0.188*	0.345†	
Pseudotsuga menziesii	0.433*	0.72*	0.937*	0.458*	0.478*	
Quercus chrysolepis	0.669*	0.857*	0.709*	0.274*	0.435*	
Quercus kelloggii	0.431	0.847†	0.59†	0.175*	0.415	

Supplementary Table 3.2 Results table for reanalysis with Mean Temperature of the Warmest Month, Mean Summer Precipitation, and Mean Winter Precipitation. Values appended with * indicate statistical significance ($p < .05$), and † denotes $p < .1$. Schoener's D and Centroid Distance were tested for statistical significance using the one-sided niche equivalency test and two-sided Hotelling's T2 Test, respectively (*Chrysolepis chrysophylla*: $p_{Schoener's\ D,\ Burned} = .215$, $p_{Schoener's\ D,\ Unburned} = .00398$, $p_{Centroid\ Distance,\ Burned} = .118$, $p_{Centroid\ Distance,\ Unburned} < 2.20e^{-16}$; *Picea engelmannii*: $p_{Schoener's\ D,\ Burned} = .047$, $p_{Schoener's\ D,\ Unburned} = .00398$, $p_{Centroid\ Distance,\ Burned} = .0499$, $p_{Centroid\ Distance,\ Unburned} < 2.20e^{-16}$; *Pinus contorta*: $p_{Schoener's\ D,\ Burned} = .115$, $p_{Schoener's\ D,\ Unburned} = .00398$, $p_{Centroid\ Distance,\ Burned} = .235$, $p_{Centroid\ Distance,\ Unburned} < 2.20e^{-16}$; *Pinus ponderosa*: $p_{Schoener's\ D,\ Burned} = .127$, $p_{Schoener's\ D,\ Unburned} = .00398$, $p_{Centroid\ Distance,\ Burned} = 6.89e^{-5}$, $p_{Centroid\ Distance,\ Unburned} = 5.27e^{-10}$; *Pseudotsuga menziesii*: $p_{Schoener's\ D,\ Burned} = .00398$, $p_{Schoener's\ D,\ Unburned} = .00398$, $p_{Centroid\ Distance,\ Burned} = 3.10e^{-4}$, $p_{Centroid\ Distance,\ Unburned} < 2.20e^{-16}$; *Quercus chrysolepis*: $p_{Schoener's\ D,\ Burned} = .00398$, $p_{Schoener's\ D,\ Unburned} = .00398$, $p_{Centroid\ Distance,\ Burned} = 5.04e^{-6}$, $p_{Centroid\ Distance,\ Unburned} = 1.71e^{-7}$; *Quercus kelloggii*: $p_{Schoener's\ D,\ Burned} = .406$, $p_{Schoener's\ D,\ Unburned} = .0518$, $p_{Centroid\ Distance,\ Burned} = .00823$, $p_{Centroid\ Distance,\ Unburned} = .00686$). $CD_B - CD_U$ is the result of subtracting the Centroid Distance in unburned samples by the Centroid Distance in burned samples, where the null hypothesis is that $CD_B - CD_U = 0$ and wildfire occurrence does not impact SORD. Statistical significance for this metric was calculated using a two-sided bootstrap test. The sample sizes used to produce these metrics are found in Table 1.

	Schoener's D		Centroid Distance		Difference in Centroid Distance (CD^B - CD^U)	
	Burned	Unburned	Burned	Unburned	Mean	95% CI
Chrysolepis chrysophylla	0.566*	0.648*	0.561	0.74*	-0.179	
Picea engelmannii	0.498	0.776*	0.513†	0.389*	0.124	
Pinus albicaulis	0.319	0.777*	0.411	0.334*	0.077	
Pinus contorta	0.529	0.7*	0.332	0.355*	-0.023	
Pinus ponderosa	0.485	0.704*	0.767*	0.251*	0.516	
Pseudotsuga menziesii	0.429*	0.714*	1.309*	0.702*	0.607*	
Quercus chrysolepis	0.683*	0.864*	0.821*	0.348*	0.472*	
Quercus kelloggii	0.611	0.835*	0.784	0.276*	0.508	

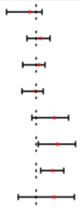

Supplementary Table 3.3 Results table for reanalysis with Mean Temperature of the Warmest Month, Mean Temperature of Coldest Month, Mean Summer Precipitation, Mean Winter Precipitation, and Potential Evapotranspiration. Values appended with * indicate statistical significance ($p < .05$), and † denotes $p < .1$. Schoener's D and Centroid Distance were tested for statistical significance using the one-sided niche equivalency test and two-sided Hotelling's T2 Test, respectively (*Chrysolepis chrysophylla*: $p_{\textit{Schoener's D}, \text{Burned}} = .0319$, $p_{\textit{Schoener's D}, \text{Unburned}} = .00398$, $p_{\text{Centroid Distance, Burned}} = .139$, $p_{\text{Centroid Distance, Unburned}} < 2.20e^{-16}$; *Picea engelmannii*: $p_{\textit{Schoener's D}, \text{Burned}} = .112$, $p_{\textit{Schoener's D}, \text{Unburned}} = .00398$, $p_{\text{Centroid Distance, Burned}} = .0729$, $p_{\text{Centroid Distance, Unburned}} < 2.20e^{-16}$; *Pinus albicaulis*: $p_{\textit{Schoener's D}, \text{Burned}} = .322$, $p_{\textit{Schoener's D}, \text{Unburned}} = .00398$, $p_{\text{Centroid Distance, Burned}} = .193$, $p_{\text{Centroid Distance, Unburned}} = < 2.20e^{-16}$; *Pinus contorta*: $p_{\textit{Schoener's D}, \text{Burned}} = .195$, $p_{\textit{Schoener's D}, \text{Unburned}} = .00398$, $p_{\text{Centroid Distance, Burned}} = .320$, $p_{\text{Centroid Distance, Unburned}} < 2.20e^{-16}$; *Pinus ponderosa*: $p_{\textit{Schoener's D}, \text{Burned}} = .139$, $p_{\textit{Schoener's D}, \text{Unburned}} = .00398$, $p_{\text{Centroid Distance, Burned}} = 6.11e^{-5}$, $p_{\text{Centroid Distance, Unburned}} = 4.09e^{-11}$; *Pseudotsuga menziesii*: $p_{\textit{Schoener's D}, \text{Burned}} = .00398$, $p_{\textit{Schoener's D}, \text{Unburned}} = .00398$, $p_{\text{Centroid Distance, Burned}} = .00129$, $p_{\text{Centroid Distance, Unburned}} < 2.20e^{-16}$; *Quercus chrysolepis*: $p_{\textit{Schoener's D}, \text{Burned}} = .00398$, $p_{\textit{Schoener's D}, \text{Unburned}} = .00797$, $p_{\text{Centroid Distance, Burned}} = 2.35e^{-5}$, $p_{\text{Centroid Distance, Unburned}} = 4.99e^{-7}$; *Quercus kelloggii*: $p_{\textit{Schoener's D}, \text{Burned}} = .797$, $p_{\textit{Schoener's D}, \text{Unburned}} = .0145$, $p_{\text{Centroid Distance, Burned}} = .00531$, $p_{\text{Centroid Distance, Unburned}} = .00156$). $\text{CD}_\text{B} - \text{CD}_\text{U}$ is the result of subtracting the Centroid Distance in unburned samples by the Centroid Distance in burned samples, where the null hypothesis is that $\text{CD}_\text{B} - \text{CD}_\text{U} = 0$ and wildfire occurrence does not impact SORD. Statistical significance for this metric was calculated using a two-sided bootstrap test. The sample sizes used to produce these metrics are found in Table 1.

Results with Different Sample Size Minima

	Schoener's D		Centroid Distance		Difference in Centroid Distance (CD^B - CD^U)	
	Burned	Unburned	Burned	Unburned	Mean	95% CI
**N ≥ 5**						
Chrysolepis chrysophylla	0.518*	0.641*	0.529	0.677*	-0.148	
Picea engelmannii	0.369*	0.751*	0.508†	0.375*	0.132	
Pinus albicaulis	0.32	0.768*	0.294	0.234*	0.06	
Pinus contorta	0.534	0.692*	0.308	0.314*	-0.006	
Pinus ponderosa	0.498	0.68*	0.616*	0.198*	0.418	
Pseudotsuga menziesii	0.421*	0.704*	1.095*	0.592*	0.504*	
Quercus chrysolepis	0.688*	0.852*	0.714*	0.282*	0.432*	
Quercus kelloggii	0.574	0.825*	0.641†	0.194*	0.447	
**N ≥ 10**						
Chrysolepis chrysophylla	0.518*	0.641*	0.529	0.677*	-0.148	
Picea engelmannii	0.369*	0.751*	0.508†	0.375*	0.132	
Pinus contorta	0.534	0.692*	0.308	0.314*	-0.006	
Pinus ponderosa	0.498	0.68*	0.616*	0.198*	0.418	
Pseudotsuga menziesii	0.421*	0.704*	1.095*	0.592*	0.504*	
Quercus chrysolepis	0.688*	0.852*	0.714*	0.282*	0.432*	
**N ≥ 25**						
Chrysolepis chrysophylla	0.518*	0.641*	0.529	0.677*	-0.148	
Pinus contorta	0.534	0.692*	0.308	0.314*	-0.006	
Pinus ponderosa	0.498	0.68*	0.616*	0.198*	0.418	
Pseudotsuga menziesii	0.421*	0.704*	1.095*	0.592*	0.504*	
Quercus chrysolepis	0.688*	0.852*	0.714*	0.282*	0.432*	

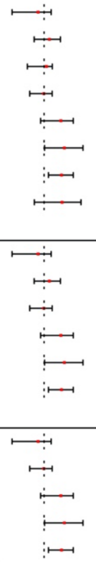

Supplementary Table 4 Results table showing the primary SORD analysis using different thresholds of minimum plot sample size. Values appended with * or † indicate statistically significant evidence of SORD ($p < .05$ and $p < .1$, respectively), where the null hypothesis of no SORD corresponds to Schoener's D = 1 and Centroid Distance = 0. The sample sizes used to produce these metrics are found in Table 1. The results with N ≥ 5 are presented in the manuscript, and N ≥ 10 and N ≥ 25 results confirm that the observation that wildfire is correlated with greater SORD for some species is consistent across different minimum sample sizes.

Supplementary Figures

Climate-space PCA of Seedling-Only and Tree-Plus-Seedling Plots by Species

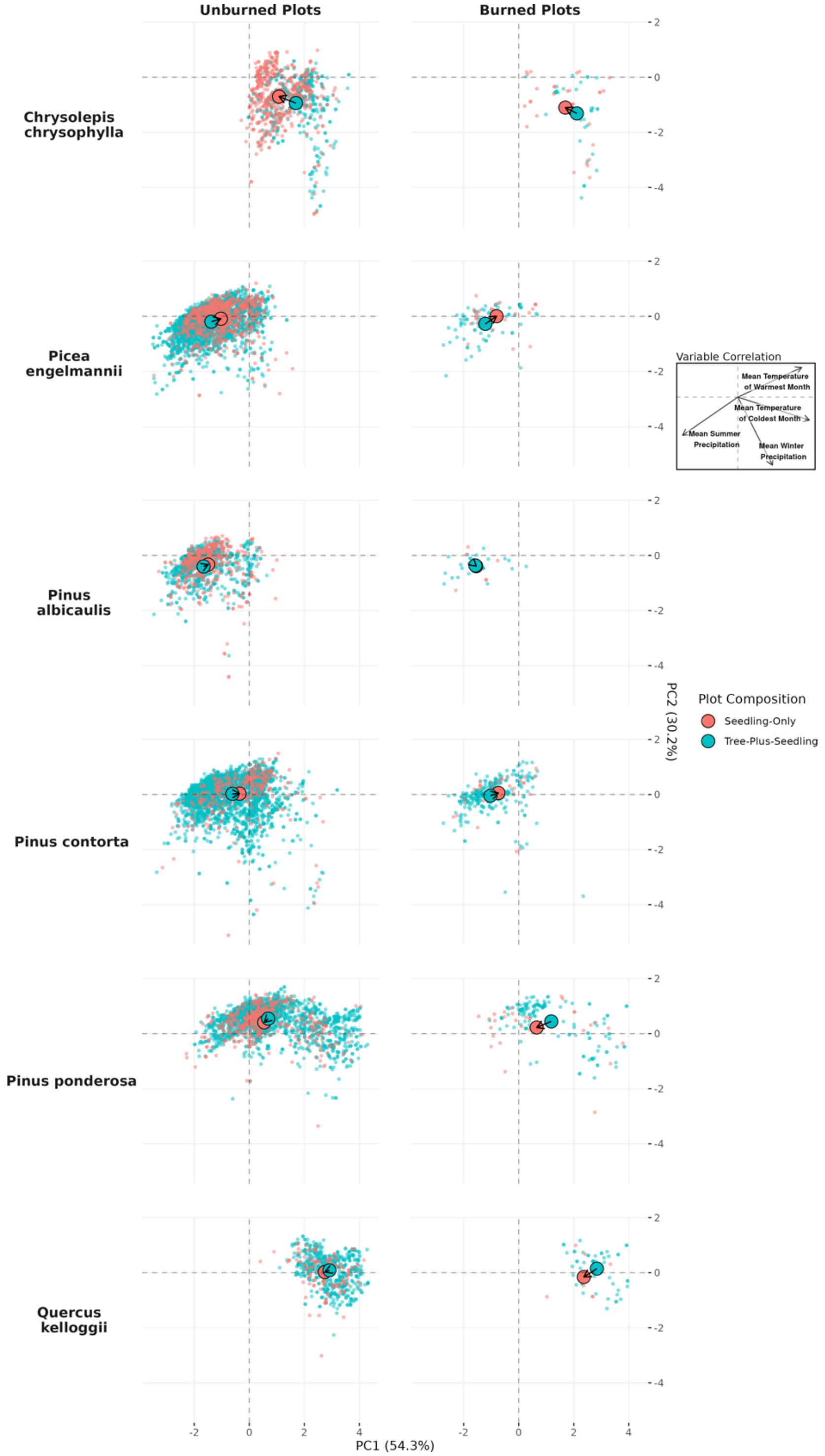

Supplementary Figure 1 Scaled PCA plots of the climatic niches of the seedling-only and tree-plus-seedling plots of each species used in the primary analysis. Centroids are shown as large circles. The first two principal components explain 84.5% of the variation.

Average Differences in the Climatic Niche Components for each Species

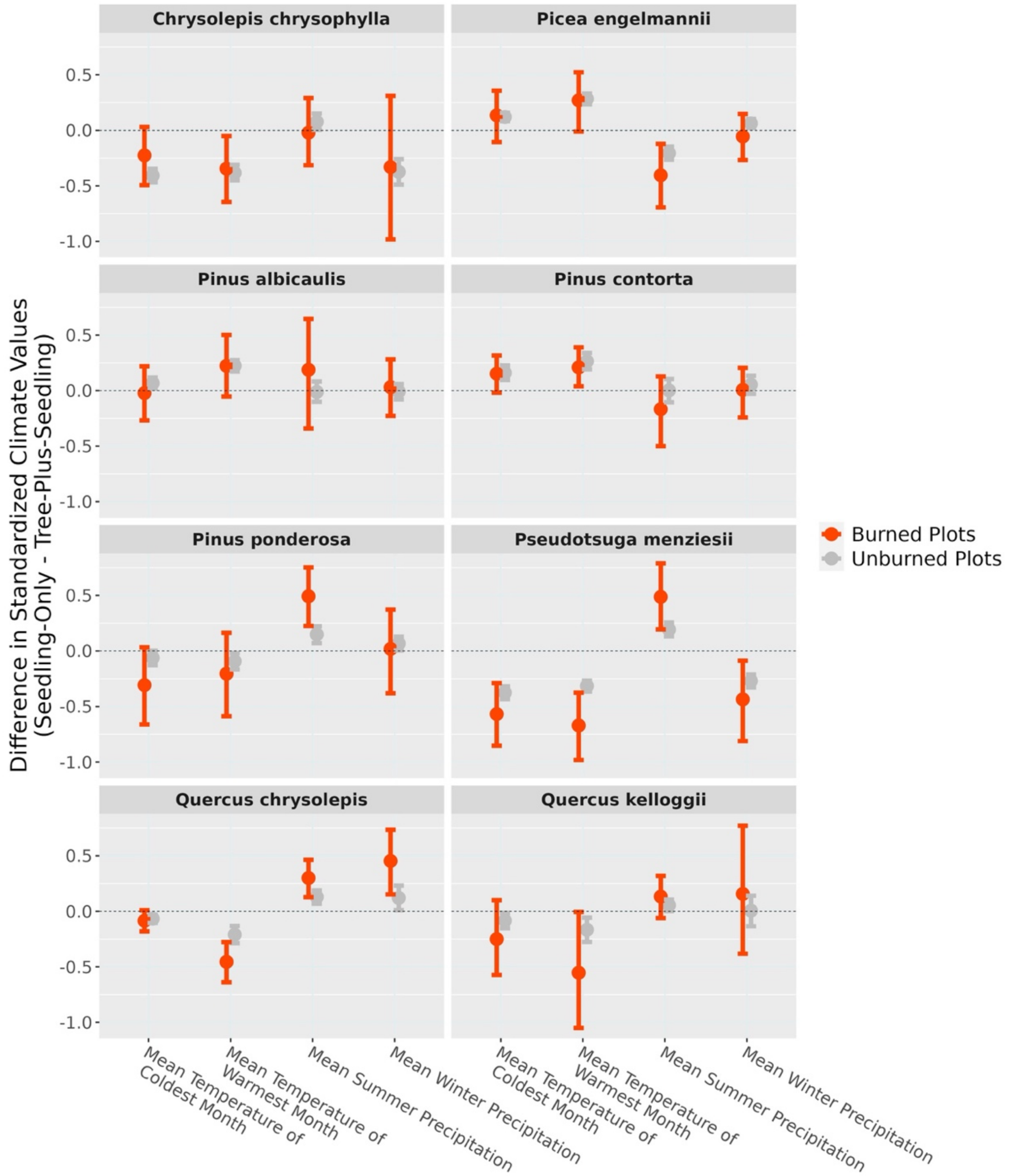

Supplementary Figure 2 SORD separated into its constituent climatic variables for each species. Data are presented as mean values ± 95% confidence intervals which were calculated using 3000 bootstraps. The sample sizes used to produce these metrics are found in Table 1. This figure shows the Seedling Only Range Displacements (SORDs) for each species, broken down into their constituent climatic variables. The interpretation of this figure is enriched by Figure 5 in the main text, which shows that the study area experienced an increase in mean temperatures of the warmest and coldest months, and a decrease in both winter and summer precipitation between 1961 and 2010. With notable exceptions among the subalpine species *Picea engelmannii*, *Pinus contorta*, and *Pinus albicaulis*, the seedling-only populations of most species occupied cooler and wetter climatic conditions.

SORD Broken Down into its Constituent Climatic Variables and Aggregated Across Species

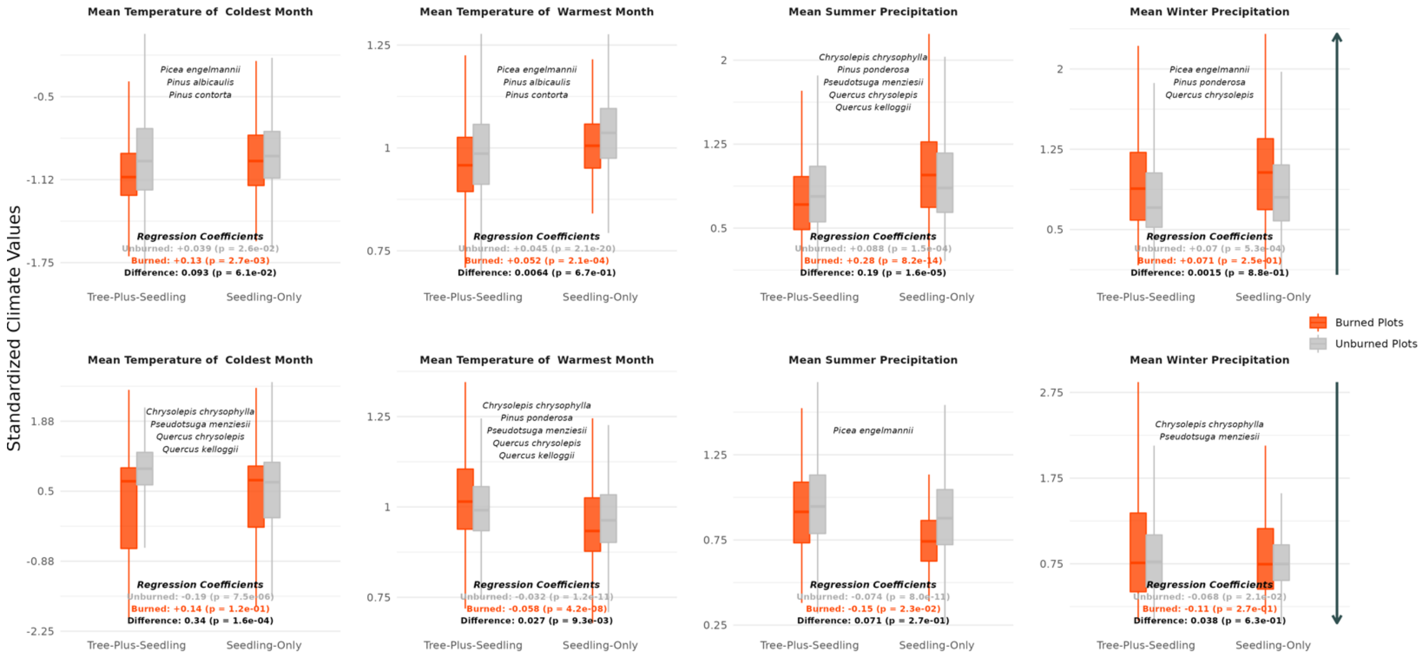

Supplementary Figure 3 SORD broken down into its constituent climatic variables, separated by wildfire history, and aggregated across species. The plot includes results for all species that share the direction of a statistically significant SORD in unburned plots (two-sided t-test, $p < .05$; $n_{MTCM\uparrow} = 3{,}919$, $n_{MTCM\downarrow} = 2{,}985$, $n_{MTWM\uparrow} = 3{,}919$, $n_{MTWM\downarrow} = 3{,}731$, $n_{MSP\uparrow} = 3{,}731$, $n_{MSP\downarrow} = 3{,}994$, $n_{MWP\uparrow} = 5{,}449$, $n_{MWP\downarrow} = 2{,}105$). Boxplots include the median line, a box denoting the interquartile range, and whiskers showing values ± 1.5x the interquartile range. Climate variables were standardized by dividing the values by their root-mean-squares. Multiple linear regression was used to quantify the difference in SORD between burned and unburned samples.

Testing for Correlation Between Slope Aspect and Fire Occurrence Across Study Area

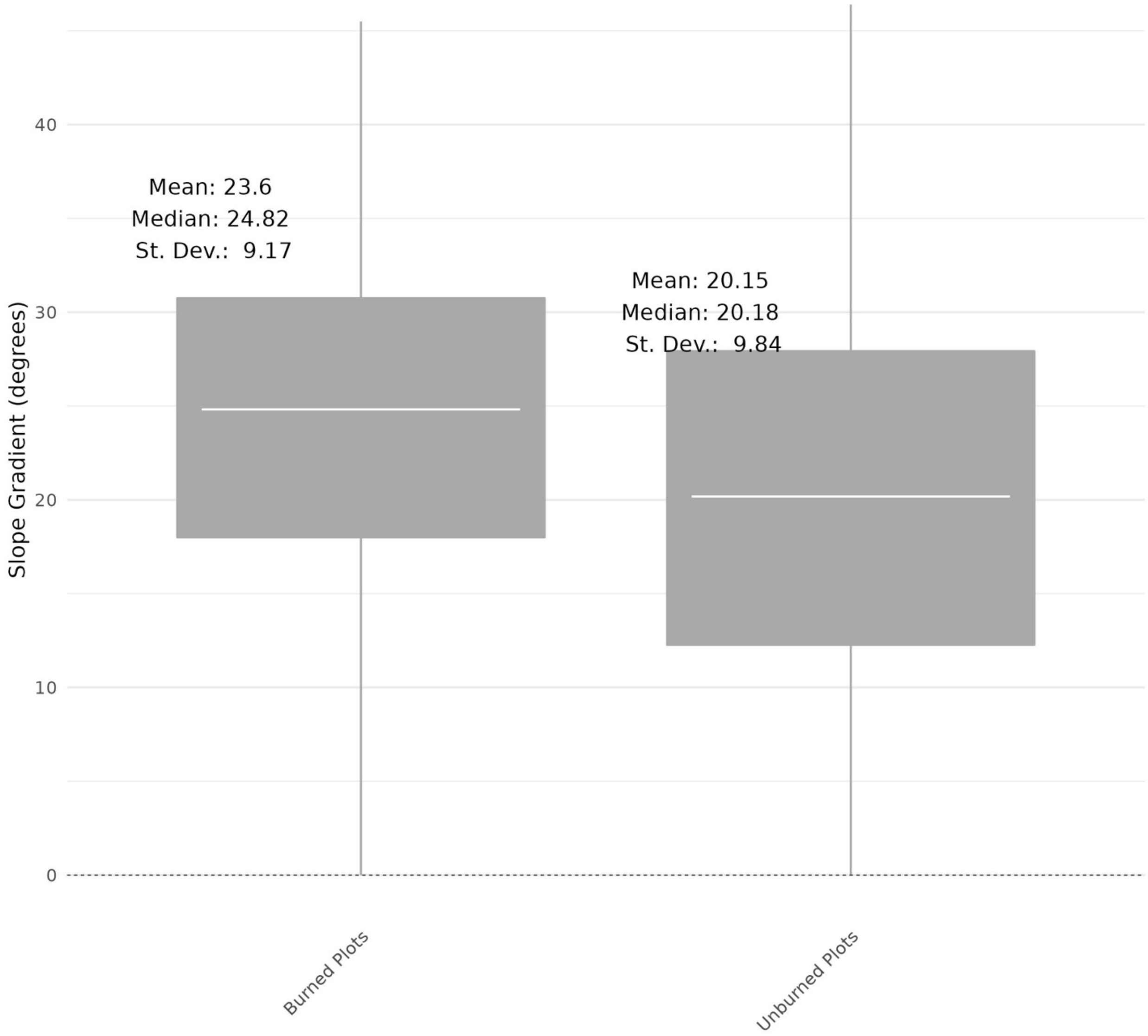

Supplementary Figure 4 Boxplot showing the relationship between slope gradient and fire occurrence across the study area (n_{plots} = 10,160). Boxplots include the median line, a box denoting the interquartile range, and whiskers denoting values within ± 1.5x the interquartile range. A two-sided t-test showed that there was a statistically significant positive correlation between slope gradient and fire occurrence ($p < 2.2e^{-16}$).

Supplementary References

1. Early, R. & Sax, D. F. Climatic niche shifts between species' native and naturalized ranges raise concern for ecological forecasts during invasions and climate change: Niche shift during naturalization. *Global Ecology and Biogeography* **23**, 1356–1365 (2014).
2. Petitpierre, B. *et al.* Climatic Niche Shifts Are Rare Among Terrestrial Plant Invaders. *Science* **335**, 1344–1348 (2012).
3. Naimi, B. & Araújo, M. B. sdm: a reproducible and extensible R platform for species distribution modelling. *Ecography* **39**, 368–375 (2016).
