# Supplementary material for: Forest fires and climate-induced tree range shifts in the western US
Source: Nat Commun. 2021 Nov 15;12:6583. doi: 10.1038/s41467-021-26838-z (PMC8594433; doi:10.1038/s41467-021-26838-z)
Supplement: Supplementary file 3 — Reporting Summary [file 41467_2021_26838_MOESM3_ESM.pdf]

Corresponding author(s): Avery P Hill
Last updated by author(s): Oct 13, 2021

Reporting Summary

Nature Portfolio wishes to improve the reproducibility of the work that we publish. This form provides structure for consistency and transparency in reporting. For further information on Nature Portfolio policies, see our Editorial Policies and the Editorial Policy Checklist.

Statistics

For all statistical analyses, confirm that the following items are present in the figure legend, table legend, main text, or Methods section.

n/a	Confirmed	
☐	☒	The exact sample size (*n*) for each experimental group/condition, given as a discrete number and unit of measurement
☐	☒	A statement on whether measurements were taken from distinct samples or whether the same sample was measured repeatedly
☐	☒	The statistical test(s) used AND whether they are one- or two-sided<br>*Only common tests should be described solely by name; describe more complex techniques in the Methods section.*
☐	☒	A description of all covariates tested
☐	☒	A description of any assumptions or corrections, such as tests of normality and adjustment for multiple comparisons
☐	☒	A full description of the statistical parameters including central tendency (e.g. means) or other basic estimates (e.g. regression coefficient) AND variation (e.g. standard deviation) or associated estimates of uncertainty (e.g. confidence intervals)
☐	☒	For null hypothesis testing, the test statistic (e.g. *F*, *t*, *r*) with confidence intervals, effect sizes, degrees of freedom and *P* value noted<br>*Give P values as exact values whenever suitable.*
☒	☐	For Bayesian analysis, information on the choice of priors and Markov chain Monte Carlo settings
☒	☐	For hierarchical and complex designs, identification of the appropriate level for tests and full reporting of outcomes
☒	☐	Estimates of effect sizes (e.g. Cohen's *d*, Pearson's *r*), indicating how they were calculated

Our web collection on statistics for biologists contains articles on many of the points above.

Software and code

Policy information about availability of computer code

Data collection	The FIA DataMart webapp tool (version 8.0, https://apps.fs.usda.gov/fia/datamart/) was used to access the forest inventory records.
Data analysis	RStudio (v 1.1.463), R (v 4.1.0) were used to execute scripts written by the authors (available https://github.com/avephill/wildfire-plant_RS) and one script written by Broennimann et al. 2011 (https://onlinelibrary.wiley.com/action/downloadSupplement?doi=10.1111%2Fj.1466-8238.2011.00698.x&file=GEB_698_sm_AppendixS1.zip)

For manuscripts utilizing custom algorithms or software that are central to the research but not yet described in published literature, software must be made available to editors and reviewers. We strongly encourage code deposition in a community repository (e.g. GitHub). See the Nature Portfolio guidelines for submitting code & software for further information.

Data

Policy information about availability of data

All manuscripts must include a data availability statement. This statement should provide the following information, where applicable:

- Accession codes, unique identifiers, or web links for publicly available datasets
- A description of any restrictions on data availability
- For clinical datasets or third party data, please ensure that the statement adheres to our policy

The climate and plant occurrence data used to support the findings of this study are available from the AdaptWest Project (https://adaptwest.databasin.org/) and Forest Inventory Analysis (http://apps.fs.usda.gov/fia/datamart), respectively. Source data are provided with this paper.

Field-specific reporting

Please select the one below that is the best fit for your research. If you are not sure, read the appropriate sections before making your selection.

☐ Life sciences ☐ Behavioural & social sciences ☒ Ecological, evolutionary & environmental sciences

For a reference copy of the document with all sections, see nature.com/documents/nr-reporting-summary-flat.pdf

Ecological, evolutionary & environmental sciences study design

All studies must disclose on these points even when the disclosure is negative.

Study description	We used all FIA plots (74,069 total) within the Northwestern Forested Mountains and Marine West Coast Forest ecoregions of the continental U.S. for a natural experiment, with historic wildfires as the treatment and observed tree migration rate as the response.
Research sample	CSV files containing forest demographic data and site conditions for 74,069 FIA plots were downloaded using the FIA DataMart tool (version 8.0, https://apps.fs.usda.gov/fia/datamart). Raster files for 19 bioclimatic climate variables were downloaded from the AdaptWest Project (https://adaptwest.databasin.org/)
Sampling strategy	All plots provided by the FIA that had information on fire occurrence and exhaustive species lists were sampled, as were all corresponding climate data for these plots.
Data collection	U.S. Forest Service crews provide the occurrence data used in this study. Phase 1 of data collection utilizes remote sensing to determine the extent of forested land to survey. In Phase 2, ground crews establish plots every ~6,000 acres of forest and tally tree species, seedling species, burned status, and many other forest metrics not used in this study.
Timing and spatial scale	The FIA plant occurrence data used were collected between 1999 and 2019. At the time of data accession, this represented all data collected under a consistent methodology. Climate data observations from 1981-2010 were downscaled and ensembled in 2016. These climate data were downloaded at the extent of North America but cropped to the study area before use.
Data exclusions	Tree species without sufficient sample size for climatic niche modeling were excluded from further analysis. Our minimum required sample size was 6 occurrences in each of 4 independent groups of occurrences for a species. Tree species were further vetted from primary analysis if consistency in the direction of potential range shift was not exhibited.
Reproducibility	Upon downloading the publicly available data sources referenced, analysis is reproducible using the code provided by the authors on GitHub.
Randomization	We sourced all FIA plots within the study area and allocated the plots by species composition, species life stage, and burned status. No randomization was used because we used all available plot data and climate data within the study area.
Blinding	Our study did not have human subjects so blinding is not relevant.

Did the study involve field work? ☐ Yes ☒ No

Reporting for specific materials, systems and methods

We require information from authors about some types of materials, experimental systems and methods used in many studies. Here, indicate whether each material, system or method listed is relevant to your study. If you are not sure if a list item applies to your research, read the appropriate section before selecting a response.

Materials & experimental systems

n/a	Involved in the study
☒	☐ Antibodies
☒	☐ Eukaryotic cell lines
☒	☐ Palaeontology and archaeology
☒	☐ Animals and other organisms
☒	☐ Human research participants
☒	☐ Clinical data
☒	☐ Dual use research of concern

Methods

n/a	Involved in the study
☒	☐ ChIP-seq
☒	☐ Flow cytometry
☒	☐ MRI-based neuroimaging
